# Hybrid photonic crystal fiber in chemical sensing

**DOI:** 10.1186/s40064-016-2415-y

**Published:** 2016-06-16

**Authors:** Sayed Asaduzzaman, Kawsar Ahmed, Touhid Bhuiyan, Tanjila Farah

**Affiliations:** Department of Information and Communication Technology (ICT), Mawlana Bhashani Science and Technology University (MBSTU), Santosh, Tangail, 1902 Bangladesh; Department of Software Engineering, Daffodil International University, Dhaka, Bangladesh; Department of Electrical and Computer Engineering, North South University, Dhaka, Bangladesh

**Keywords:** Birefringence, Elliptical hole, Sensitivity, Confinement loss, Lower indexed chemical sensor, Hybrid photonic crystal fiber

## Abstract

**Background:**

In this article, a hybrid photonic crystal fiber has been proposed for chemical sensing. A FEM has been applied for numerical investigation of some propagation characteristics of the PCF at a wider wavelength from 0.7 to 1.7 µm. The geometrical parameters altered to determine the optimized values. The proposed PCF contains three rings of circular holes in the cladding where the core is formulated with microstructure elliptical holes.

**Results:**

The simulation result reveals that our proposed PCF exhibits high sensitivity and low confinement loss for benzene, ethanol and water than the prior PCFs. We have also shown that our proposed PCF shows high birefringence for benzene 1.544 × 10^−3^, for ethanol 1.513 × 10^−3^ and for water 1.474 × 10^−3^ at λ = 1.33 µm.

**Conclusion:**

The proposed PCF is simple with three rings which can be used for the sensing applications of industrially valuable lower indexed chemicals.

## Background

Fiber optic technology is not bounded in just telecommunication purposes as it was first excogitated. Day by day new applications of optical fiber has been emerged. Photonic crystal fiber broadens the applications of optical fiber not only in communication but also in wide areas by diminishing the limitations of the conventional fibers. In photonic crystal fiber, a bunch of tiny microscopic air holes remains along the entire fiber (Russell 2003; Knight 2003). Index guiding PCF and photonic band gap PCF are the two kinds of PCFs. In photonic band gap PCF the light is guided by photonic band gap mechanism where the core is large air core (Fini 2004). Another type of PCF is index guiding PCF where the core is solid having a higher refractive index than the cladding part (Hoo et al. 2003; Monro et al. 2001). For some unique and exceptional features PCF has been used for nonlinear optics (Ebendorff-Heidepriem et al. 2004), optical coherence tomography (Humbert et al. 2006), high-power technology (Lecaplain et al. 2010), multi wavelength generation (Pinto et al. 2011), super continuum generation (Dudley et al. 2006) and spectroscopy (Holzwarth et al. 2000).

In recent years due to the advancement of technology the PCFs are used for sensing of toxic and harmful gases (Morshed et al. 2015a, b, c), chemicals (Ademgil 2014; Park et al. 2011) and biomedical (Jensen et al. 2005; Dinish et al. 2012) applications. The sensing mechanism takes place by detecting the analytes filled holes in the core region with the evanescent field through the interaction of lights. The interactive features of PCF enable to observe the guiding properties like birefringence (Habib et al. 2013), dispersion (Begum et al. 2009) and nonlinearity (Habib et al. 2014). The first designed PCF was hexagonal (Knight et al. 1996) shaped but due to the autonomous geometrical structures and the advancement of technology numerous PCFs have been designed. By applying different geometrical structure like octagonal (Ademgil 2014), decagonal (Razzak et al. 2007), elliptical (Hao et al. 2013), honey comb cladding (Hou et al. 2013), hybrid cladding (Morshed et al. 2015a) better guiding properties have been achieved.

In the article (Ademgil 2014), a new hexagonal and octagonal PCF has been used for benzene, ethanol and water sensing where the core was microstructure. An octagonal shape PCF (O-PCF) with five rings in cladding has been proposed which shows higher sensitivity and lower confinement loss (Ahmed and Morshed 2016). The O-PCF was a modified structure of the article (Ademgil 2014) where there were three rings in the cladding. Asaduzzaman et al. (2015a, b, c) proposed different PCF structures, where the holes in the core region were elliptical. Ademgil and Haxha (2015) proposed two new PCF structure with vertically and horizontally arranged elliptical air holes for both core and cladding and got high sensitivity, high birefringence and low confinement loss. The concept of filling the core/cladding of the PCFs with various analytes drew much attention of researchers during the last decades (Kuhlmey et al. 2009). By filling holes with analytes different chemicals and biological substances can be sensed. In recent years researchers has been working to improve the optical properties of PCF. Elliptical holes were used to get higher birefringence (Qiu and He 1999; Yue et al. 2007).

In this paper, a Hybrid PCF (H-PCF) has been proposed which shows high birefringence lower confinement loss and also shows high sensitivity for three analytes benzene, water and ethanol. A circular PCF is also proposed to make a comparative analysis with the proposed PCF. Our proposed hybrid PCF contains elliptical holes in the core region with three circular rings in the cladding. The geometrical parameters varied to optimize for both core and cladding. The proposed PCF shows better sensitivity and confinement loss than the previous PCFs (Ademgil 2014; Asaduzzaman et al. 2015a; Ademgil and Haxha 2015).

## Geometrical structure of the proposed H-PCF

**Fig. 1:**
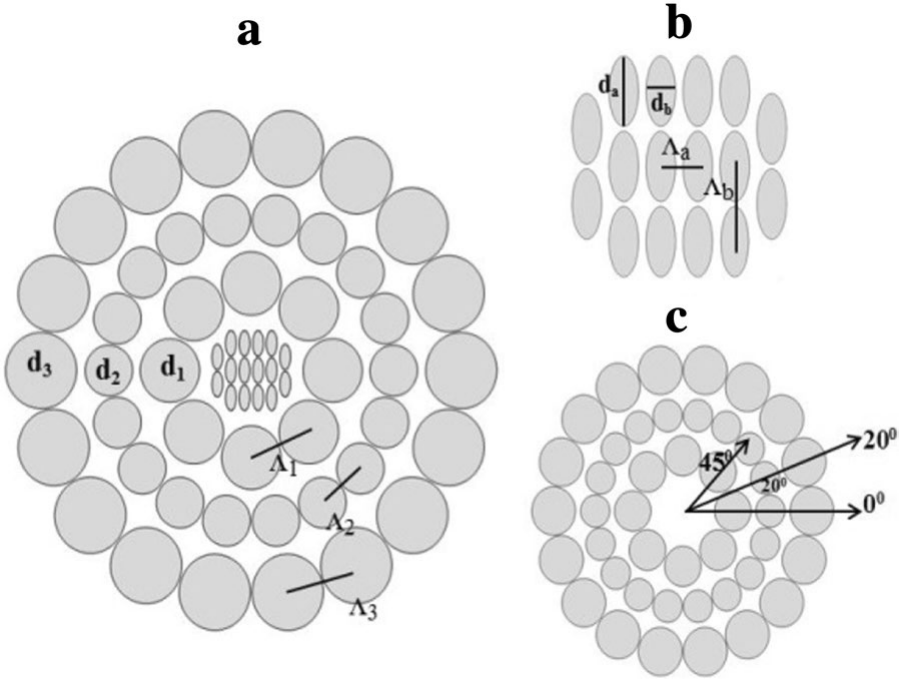
Transverse cross sectional view of **a** the proposed H-PCF, **b** core region and **c** outer cladding region with rotational angle with respect to 0° of air holes in a ring

Figure 1a shows the cross sectional view of the proposed PCF. The orientation of core region and outer cladding region with rotational angle with respect to 0° of holes in a ring has been shown in Fig. 1b, c respectively. From the figure it is clear that the proposed PCF is circular shaped with three rings of holes in the cladding region where the first, second and third ring of hole includes 8, 18 and 18 air holes respectively. The arrangements of the diameters of the second and third rings were kept, such as the cladding holds bigger air holes to make the proper interaction of light through the core. The diameters of the first, second and third rings were assumed as d_1_, d_2_ and d_3_. The distance between the centres of two adjacent holes is called pitch. The hole to hole distance of the two adjacent air holes of the first, second and third ring is denoted by Λ_1_, Λ_2_ and Λ_3_. In the article (Ademgil and Haxha 2015) the proposed PCF contains elliptical air holes for which the sensitivity increase rapidly than the previous PCFS. Our proposed H-PCF contains bunch of 16 elliptical air holes in the core where the major axis and minor axis of the elliptical holes was assumed as d_a_ and d_b_. The pitch between the two adjacent holes are denoted as Λ_a_ and Λ_b_. Figure 1c illustrates that the rotational angle of the first ring is 45° (contains 8 air holes), for both second and third ring is 20° (both contains 18 air holes) with respect to 0° angle. In our proposed PCF the thickness of the perfectly matched layer (PML) is set around 10 % between the inner and outer portion having a silica background where the refractive index of silica varies according Sellmeier’s equation.

Recently, PCFs structures were proposed with hybrid cladding in shape formation and different diameters of holes in different rings which shows better optical properties (Yuan et al. 2011; Hasan et al. 2014; Razzak and Namihira 2008). In Ademgil (2014), comparison between two PCFs (hexagonal and octagonal shape) was shown for sensing applications. The confinement loss or leakage loss can strongly be affected by the diameters of the holes in the two outermost rings in cladding (Olyaee and Naraghi 2013). The confinement, loss is decreased with the increase of the diameter of the holes. For lower confinement, loss the diameters of the holes of outermost ring were set larger than the middle layer in our proposed H-PCF. The innermost ring is responsible for sensitivity (Asaduzzaman et al. 2015a, b, c). So we kept the air holes of the innermost ring larger to gain high sensitivity as well as other guiding properties.

**Fig. 2:**
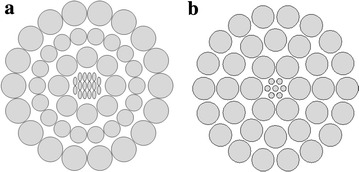
Transverse cross sectional view of **a** proposed H-PCF and **b** C-PCF

A circular shape PCF was also proposed in this paper to make a comparative analysis and to justify the design optimization of the Proposed H-PCF. The proposed circular PCF (C-PCF) is the simplest type of PCF where both core and cladding is circular. The core holes were filled with the same chemicals (benzene, ethanol and water). Figure 2 shows the cross sectional view of the Proposed H-PCF and C-PCF.

## Synopsis of numerical method

A full vectorial finite element method (FEM) applied to the circular perfectly matched layer (PML) to investigate the proposed H-PCF. The FEM is a powerful tool which can be used for full vectorial analysis of the different photonic waveguide devices (Monro et al. 2001). The propagation characteristics and optical properties of the leaky modes can be evaluated by using PMLs boundary condition at a wide range of wavelength from 0.7 to 1.7 µm. The background material of the proposed PCFs is made of silica. The Sellmeier equation is used to model the background material (Ademgil and Haxha 2015). The refractive index of silica varies with the variation of the wavelength according to the following equation:

1 $$n\left( \lambda \right) = \sqrt {\left( {1 + \frac{{B_{1} {\uplambda}^{2} }}{{{\uplambda}^{2} - C_{1} }} + \frac{{B_{2} {\uplambda}^{2} }}{{{\uplambda}^{2} - C_{2} }} + \frac{{B_{3} {\uplambda}^{2} }}{{{\uplambda}^{2} - C_{3} }}} \right)}$$

where, n is the refractive index of silica, λ (µm) is the wavelength, *B*_(*i*=1,2,3)_ and *C*_(*i*=1,2,3)_ are Sellmeier coefficients. The values of the Sellmeier coefficients for the fused silica have been defined in the Table 1.

**Table 1:** Values of the different Sellmeier coefficients

Sellmeier coefficient	Value
*B*_1_	0.696166300
*B*_2_	0.407942600
*B*_3_	0.897479400
*C*_1_	4.67914826 × 10^−3^ μm^2^
*C*_2_	1.35120631 × 10^−2^ μm^2^
*C*_3_	97.9340025 μm^2^

Using the anisotropic PML (Saitoh and Koshiba 2002) from Maxwell’s equations the following vectorial equation is derived as:

2 $$\nabla \times \left( {\left[ {\text{s}} \right]^{-1 }}\right)\nabla \times{\text{E}} - {\text{k}}_{0}^{2} {\text{n}}^{2} \left[ {\text{s}} \right]{\text{E}}=0$$

where, *K*_0_ = 2*π*/*λ* is the wave number in free space, n is the refractive index of the domain, E is the electric field [s] is the PML matrix, [s]^−1^ is an inverse matrix of [s] and λ is the operating wavelength. The leaking of light from the core of exterior materials results in confinement loss *L*_*c*_ which can be obtained from the imaginary part of *n*_*eff*_ using the following equation:

3 $${\text{Confinement}}\;{\text{loss}},L_{c} = \frac{40\pi }{{\ln \left( {10} \right)\lambda }}Im\left( {n_{eff} } \right) \times 10^{6} \approx 8.686K_{0} Im\left( {n_{eff} } \right) \times 10^{6} \;({\text{dB}}/{\text{m}})$$

The relative sensitivity coefficient measures the interaction between light and the material to be sensed and it can be calculated through the following equation:

4 $$r = \frac{{n_{r} }}{{n_{eff} }} f$$

where, *n*_*r*_ represents the refractive index of chemical to be sensed located in core holes and *n*_*eff*_ is the modal effective refractive index.

5 $$f = \frac{{\mathop \smallint \nolimits_{sample} Re\left( {E_{x} H_{y} - E_{y} H_{x} } \right)dxdy}}{{\mathop \smallint \nolimits_{total} Re\left( {E_{x} H_{y} - E_{y} H_{x} } \right)dxdy}} \times 100$$

The ratio *f* is the percentage of the air hole power and the total power of the H-PCF. Where, E_x_, E_y_, H_x_, and H_y_ are the transverse electric and magnetic fields of the guided mode. In PCFs, polarization maintaining (PM) properties can be used for eliminating the effect of polarization mode dispersion (PMD), stabilizing the operation of optical devices and sensing applications. Birefringence is imperative for the polarization maintaining properties (Habib et al. 2013). The birefringence is defined as:

6 $${\text{B}} = |\! {{\text{ n}}_{\text{x}} {-}{\text{n}}_{\text{y}} } |$$

where, n_x_ and n_y_ are the mode indices of the two polarizations of the fundamental modes.

## Numerical results and discussion

**Fig. 3:**
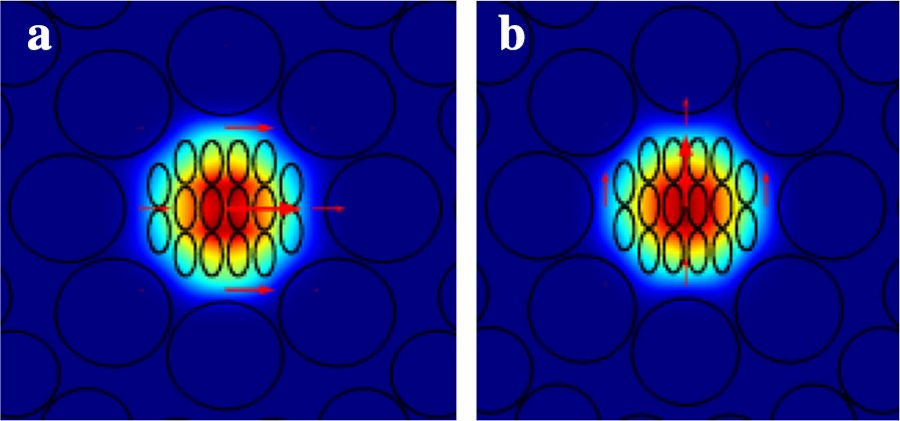
The power flow distribution of the proposed H-PCF, **a** x-polarization and **b** y-polarization for ethanol at λ = 1.33 µm

The optical properties of the proposed H-PCF according to various geometrical parameters have been analysed in this section. The overall simulation and analysis was done by filling the three analytes whose refractive index are assumed as benzene (n = 1.366), ethanol (n = 1.354) and water (n = 1.330) (Kamikawachi et al. 2008) at the elliptical holes in the core region for a wide range of wavelength from 0.7 to 1.7 µm. Figure 3 shows the power flow distribution of x-polarization and y-polarization for ethanol at an operating wavelength λ = 1.33 µm. The figure shows that the interaction of light occurs through the core region where the analyte is present. The figure also shows that the mode field is tightly confined at the core.

**Fig. 4:**
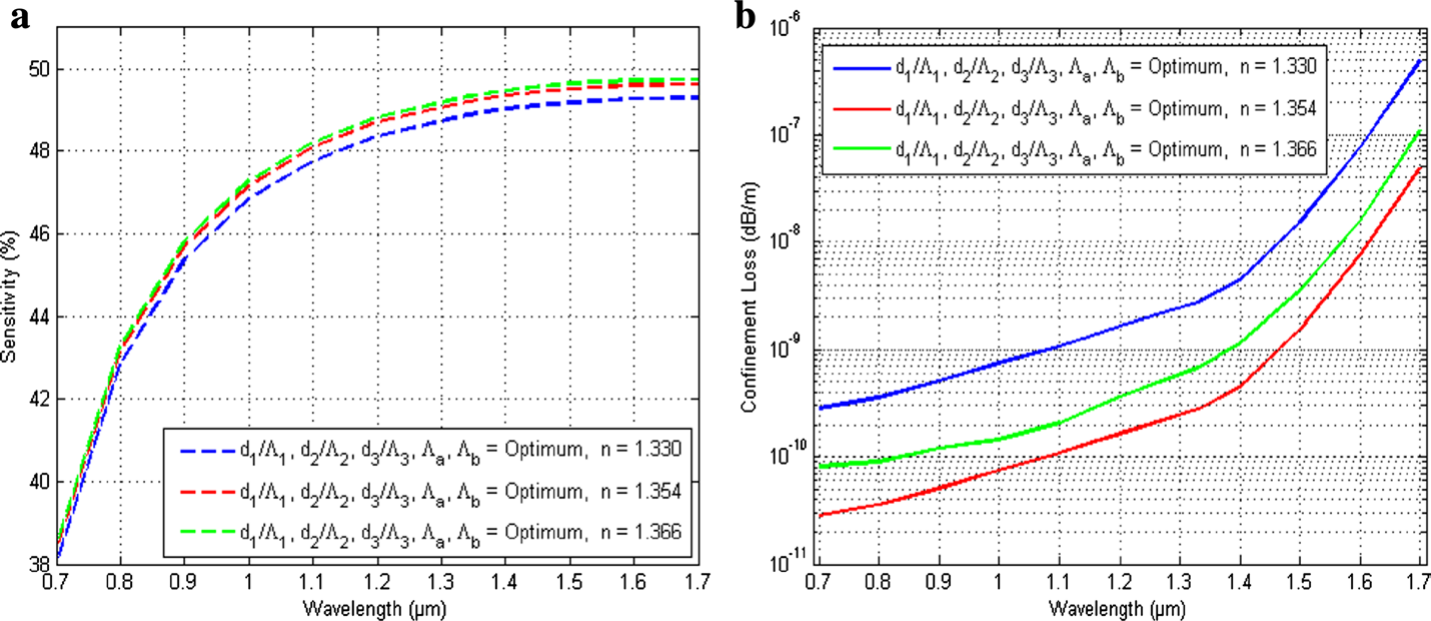
Variation of **a** sensitivity, **b** confinement loss of the proposed H-PCF for benzene (n = 1.366), ethanol (n = 1.354) and water (1.330) as a function of wavelength when optimal parameters kept constant

Figure 4 reveals the variation of Sensitivity and confinement loss of the proposed H-PCF for benzene (n = 1.366), ethanol (n = 1.354) and water (1.330) as a function of wavelength when optimal parameters d_1_/Ʌ_1_, d_2_/Ʌ_2_, d_3_/Ʌ_3_ and d_b_/d_a_ were kept constant. The geometrical parameters like d_1_/Ʌ_1_, d_2_/Ʌ_2_, d_3_/Ʌ_3_ and d_b_/d_a_ are optimized by a simple process. For these optimization process we have varies the parameters are altered. The sensitivity and confinement loss both changes with the change of operating wavelength by the Fig. 4. Both sensitivity and confinement loss increases with the increase of wavelength. For sensitivity curve it can illustrate that the sensitivity increases rapidly from 0.7 to 1.3 µm and then changes slightly. The confinement loss increases moderately from 0.7 to 1.4 µm but then increases sharply to the wavelength of 1.7 µm.

**Fig. 5:**
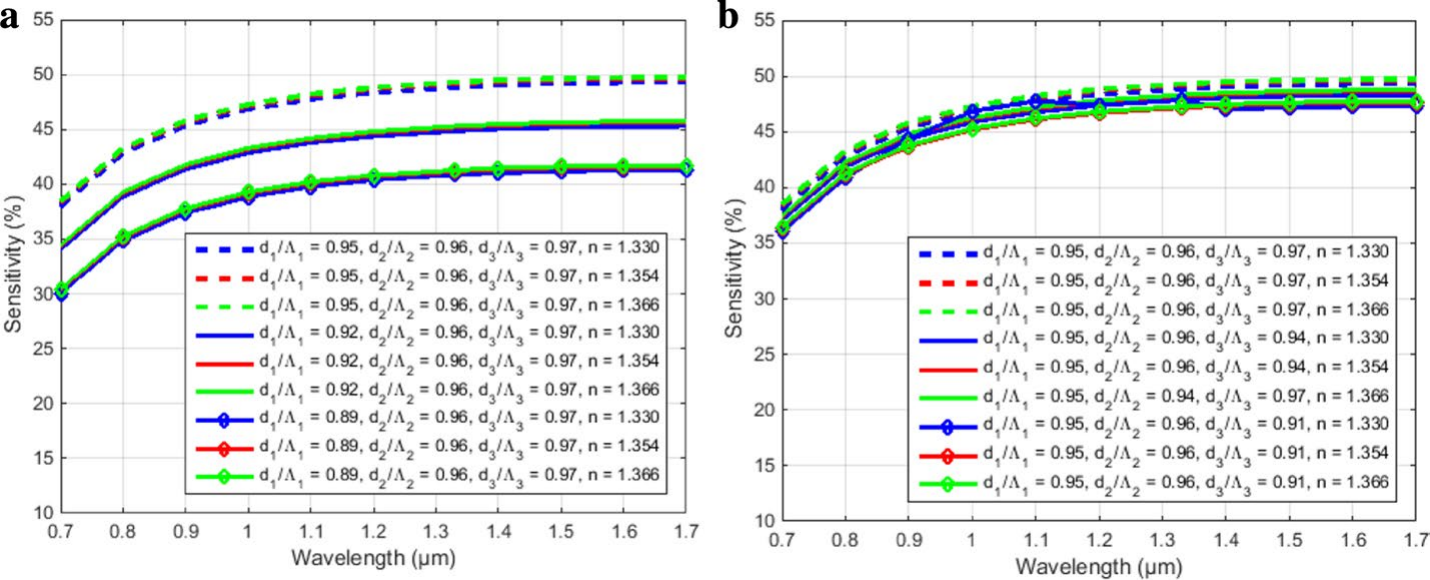
Variation of sensitivity for **a** d_1_/Ʌ_1_ = 0.95, d_1_/Ʌ_1_ = 0.92 and d_1_/Ʌ_1_ = 0.89 when d_2_/Ʌ_2_ = 0.96, d_3_/Ʌ_3_ = 0.97, d_b_/d_a_ = 0.429 were constant, **b** d_3_/Ʌ_3_ = 0.97, d_3_/Ʌ_3_ = 0.94 and d_3_/Ʌ_3_ = 0.91 when d_2_/Ʌ_2_ = 0.96, d_1_/Ʌ_1_ = 0.95, d_b_/d_a_ = 0.429 were constant as a function of wavelength for benzene (n = 1.366), ethanol (n = 1.354) and water (1.330)

Figure 5 illustrates the effect of an inner ring (d_1_/Ʌ_1_) and outer ring (d_3_/Ʌ_3_) of the cladding of proposed H-PCF. From the figure it can depict that the inner holes have a great impact on sensitivity as sensitivity decreases with the decrease of inner hole. A slight change occurs with the change of the air filling ratio of outer ring. Birefringence does not change with the change of the inner and outer ring but confinement loss changes a little bit in holes diameters of outer ring as confinement loss greatly depends on outermost ring.

**Fig. 6:**
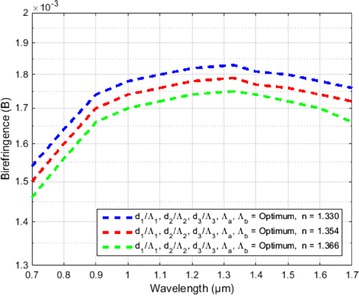
Variation of birefringence as a function of wavelength for benzene (n = 1.366), ethanol (n = 1.354) and water (1.330) when the other optimized parameters d_1_/Ʌ_1_ = 0.95, d_2_/Ʌ_2_ = 0.96, d_1_/Ʌ_1_ = 0.97, d_b_/d_a_ = 0.429 were constant

Figure 6 shows variation of Birefringence as a function of wavelength for the three analytes. Here from the figure it is clear that the curve shows three types of change. Firstly, the birefringence increases swiftly between the wavelength 0.7–0.9 µm then it increases sequentially from 0.9 to 1.3 µm and after that decreases slightly to the wavelength of 1.7 µm.

**Fig. 7:**
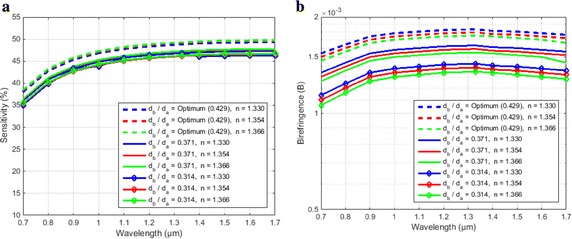
Variation of **a** sensitivity for d_b_/d_a_ = 0.429, d_b_/d_a_ = 0.371 and d_b_/d_a_ = 0.314 when d_1_/Ʌ_1_ = 0.95, d_2_/Ʌ_2_ = 0.96, d_3_/Ʌ_3_ = 0.97 were constant, **b** birefringence for d_b_/d_a_ = 0.429, d_b_/d_a_ = 0.371 and d_b_/d_a_ = 0.314 when d_1_/Ʌ_1_ = 0.95, d_2_/Ʌ_2_ = 0.96, d_3_/Ʌ_3_ = 0.97 were constant as a function of wavelength for benzene (n = 1.366), ethanol (n = 1.354) and water (n = 1.330)

The consequences of d_b_/d_a_ (ratio of elliptical holes major and minor axis) on both sensitivity and birefringence have been shown in Fig. 7. From the both figures it can be illustrate that there is a little change of sensitivity whereas a rapid change occurs with the different value of d_b_/d_a_.

**Fig. 8:**
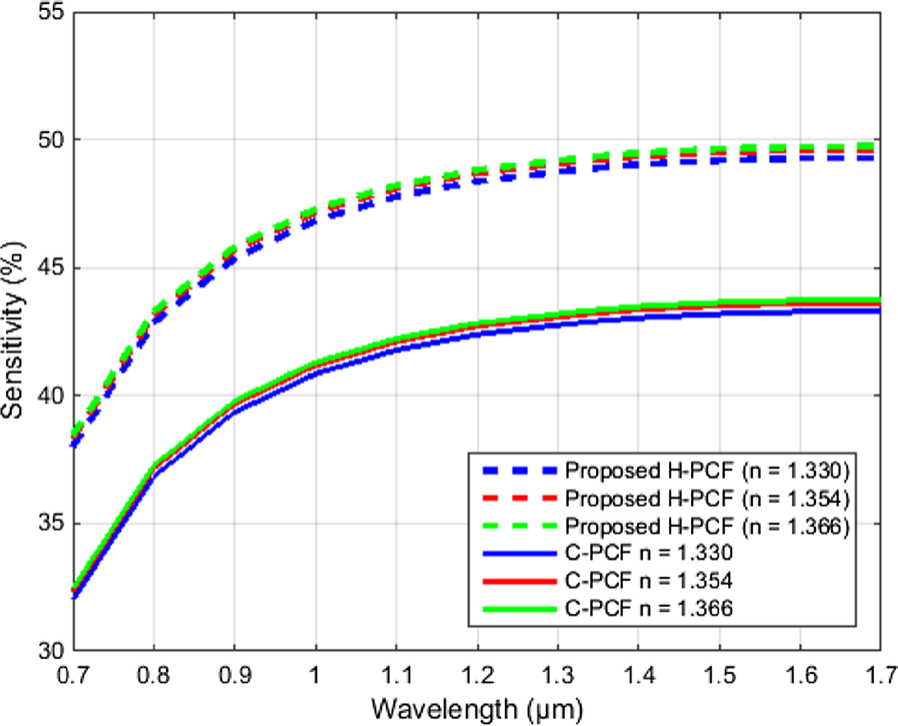
Variation of sensitivity between proposed H-PCF and C-PCF as a function of wavelength for benzene (n = 1.366), ethanol (n = 1.354) and water (n = 1.330)

We have considered a simple structure circular shaped (C-PCF) investigate the better guidance properties of our proposed H-PCF. Figure 8 exhibits that the proposed H-PCF shows higher sensitivity than the C-PCF. Besides, our proposed H-PCF shows lower confinement loss as well. Birefringence is the difference of the refractive indices of the x-polarization and y-polarization. The C-PCF does not show and significance in birefringence properties. To fabricate the Proposed H-PCF design feasibility is needed criteria. We have also investigated the H-PCF between the optimum parameters and change in global parameters of ±3 and ±6 %. Table 2 shows the comparison of sensitivity, confinement loss and birefringence among the optimum parameters and the change in global parameters for ethanol at λ = 1.33 µm. Table 3 shows the comparative analysis among the prior PCFs and the proposed H-PCF. From the table it can depict that the proposed PCF shows high sensitivity and low confinement or leakage loss than the prior PCFs. From the above discussion the optimized values of the proposed H-PCF is set as d_1_/Ʌ_1_ = 0.95 when d_2_/Ʌ_2_ = 0.96, d_3_/Ʌ_3_ = 0.97, d_b_/d_a_ = 0.429.

**Table 2:** Comparison of sensitivity, confinement loss and birefringence among the optimum parameters and the change in global parameters for ethanol at λ = 1.33 µm

Change in global parameters (%)	Sensitivity (%)	Confinement loss (dB/m)	Birefringence (B)
+6	53.07	8.94 × 10^−8^	1.603 × 10^−3^
+3	51.05	5.74 × 10^−7^	1.553 × 10^−3^
Optimum	49.17	2.75 × 10^−10^	1.513 × 10^−3^
−3	43.32	6.71 × 10^−11^	1.233 × 10^−3^
−6	38.11	1.75 × 10^−13^	1.013 × 10^−3^

**Table 3:** Comparison of simulated result and structure shape among proposed PCF and prior PCFs for ethanol at λ = 1.33 µm

PCFs	Sensitivity (%)	Confinement loss (dB/m)	No. of rings	Structural shape	
				Core	Cladding
Prior PCF_1_ (Ademgil 2014)	23.05	5.74 × 10^−6^	3	Circular hole	Circular holes in octagonal configuration
Prior PCF_2_ (Ademgil and Haxha 2015)	23.75	2.4 × 10^−4^	3	Elliptical holes	Elliptical holes in hexagonal configuration
Prior PCF_3_ (Asaduzzaman et al. 2015a)	40.32	7.55 × 10^−7^	2	Elliptical holes	Circular holes in circular configuration
Proposed H-PCF	49.17	2.75 × 10^−10^	3	Elliptical holes	Circular holes in circular configuration

Selectively filling the PCF holes with analytes is very challenging work. However due to advancement of nanotechnology several techniques has can be used to fill the PCF core or cladding with different analytes. Huang et al. (2004) proved that it is possible to fill the cladding holes or core holes with different analytes which may be used to demonstrate the functionality of the PCF applications. The filling process takes place by pressurizing the UV-curable polymer inside the PCF. Recently, it has been proved that the PCF structures filled with liquids as well as analytes in core/cladding can be fabricated by the same technique (Luo et al. 2013; Gerosa et al. 2011). Experimental demonstration and theoretical simulation of a liquid filled core based PCF was proposed by Zhang et al. (2007) for sensing application.

Our proposed H-PCF is dual shape mixing PCF as core holes are elliptical and cladding holes are circular. Fabrication of PCF is an important issue of photonic crystal fiber. Our proposed PCF may not be easy to fabricate. Our proposed H-PCF contains the different holes size in different rings. Besides our proposed H-PCF is a dual shape mixing with different core and cladding type. The main concern is the fabrication of the core region with elliptical holes. In Chen and Shen (2007) a dual shape mixed PCF (where core holes were elliptical and outer cladding holes was circular) was proposed to achieve ultrahigh birefringence. Elliptical hole based core has been successfully manufactured in the recent years (Issa et al. 2004). Some of the techniques like Stack and Draw techniques, Drilling method can be used to fabricate the PCF structures but due to the limitations of those techniques the proposed PCF may face difficulties. Recently, a capillary stacking method was proposed which can be used to fabricate the proposed H-PCF (Argyros et al. 2001). Sol–gel casting technique would be fruitful to fabricate the proposed PCF as it is mainly used to fabricate the PCFs with different size of holes (Bise and Trevor 2005). Through these considerations and due to the advancement of nanotechnology and fabrication process we strongly believe that our proposed H-PCF can be fabricated without and major difficulties.

## Conclusion

A circular PCF with three rings of air holes including the elliptical holes based microstructure core has been proposed in this article. We have shown that our proposed PCF can show higher sensitivity, higher birefringence and lower confinement loss simultaneously. The whole numerical investigation was done by full vectorial finite element method of varying the diameters and pitch values of the both core and cladding to optimize the structure. The proposed PCF shows 49.29 % sensitivity and 3.13 × 10^−10^ dB/m confinement loss for benzene, 49.17 % sensitivity and 2.75 × 10^−10^ dB/m confinement loss for ethanol and 48.85 % sensitivity and 2.75 × 10^−9^ dB/m confinement loss for water. The main focus of this work is to detect the lower indexed chemicals which are industrially valuable.
